# Enhancing the quality of antibiotic prescribing in Primary Care: Qualitative evaluation of a blended learning intervention

**DOI:** 10.1186/1471-2296-11-34

**Published:** 2010-05-07

**Authors:** Marie-Jet Bekkers, Sharon A Simpson, Frank Dunstan, Kerry Hood, Monika Hare, John Evans, Christopher C Butler, the STAR study team

**Affiliations:** 1 South East Wales Trials Unit, School of Medicine, Cardiff University, Cardiff, UK; 2 Department of Primary Care and Public Health, School of Medicine, Cardiff University, Cardiff, UK

## Abstract

**Background:**

The Stemming the Tide of Antibiotic Resistance (STAR) Educational Program aims to enhance the quality of antibiotic prescribing and raise awareness about antibiotic resistance among general medical practitioners. It consists of a seven part, theory-based blended learning program that includes online reflection on clinicians' own practice, presentation of research evidence and guidelines, a practice-based seminar focusing on participants' own antibiotic prescribing and resistance rates in urine samples sent from their practice, communication skills training using videos of simulated patients in routine surgeries, and participation in a web forum. Effectiveness was evaluated in a randomised controlled trial in which 244 GPs and Nurse Practitioners and 68 general practices participated. This paper reports part of the process evaluation of that trial.

**Methods:**

Semi-structured, digitally recorded, and transcribed telephone interviews with 31 purposively sampled trial participants analysed using thematic content analysis.

**Results:**

The majority of participants reported increased awareness of antibiotic resistance, greater self-confidence in reducing antibiotic prescribing and at least some change in consultation style and antibiotic prescribing behaviour. Reported practical changes included adopting a practice-wide policy of antibiotic prescription reduction. Many GPs also reported increased insight into patients' expectations, ultimately contributing to improved doctor-patient rapport. The components of the intervention put forward as having the greatest influence on changing clinician behaviour were the up-to-date research evidence resources, simple and effective communication skills presented in on-line videos, and presentation of the practice's own antibiotic prescribing levels combined with an overview of local resistance data.

**Conclusion:**

Participants regarded this complex blended learning intervention acceptable and feasible, and reported wide-ranging, positive changes in attitudes and clinical practice as a result of participating in the STAR Educational Program.

**Trial registration:**

Current Controlled Trials ISRCTN63355948

## Background

### Key quote

... when you start talking to people most of them don't actually want antibiotics, they just want what is best for them (GP161)

### Antibiotic resistance

Increasing antibiotic resistance is a worldwide problem[1,2]. Developing new anti-infective agents is a costly and lengthy process, with few truly new classes of antibiotics expected to be available in the near to medium future[3,4]. Antibiotic prescribing rates in the UK are almost double those in the Netherlands and compare unfavourably to many other countries worldwide[5]. Lower levels of antibiotic prescribing, if consistently implemented on a large scale, have been associated with reduced resistance[6]. Resistance is especially problematic in relation to gram-negative organisms that cause most urinary tract infections, a common problem presented by patients in primary care, and yet some GPs do not regard the problem of antibiotic resistance an important priority in their clinical practice[7]. Other GPs are concerned that not prescribing antibiotics may have a negative impact on the clinician-patient relationship[8]. Clinicians often over-estimate patients' expectations for antibiotic treatment and at the same time recognise that patients often over-estimate the benefits of antibiotic treatment for self-limiting respiratory tract infections[9,10]. The STAR Educational Program was created to address these challenges[11].

### Theoretical foundation

The STAR Program is grounded in a clear theoretical framework[12], incorporating development work from the fields of microbiology, prescribing, education, communication, and behavioural sciences[13]. It is informed particularly by the theory of planned behaviour[14,15] and social learning theory[16,17] and so addresses both the 'how' and the 'why' of change[18]. The 'why' of change is anchored in exposure to evidence and expert opinion, i.e. the presentation of prescribing and microbiological data at a practical - locally meaningful - level, and the provision of up-to-date research evidence and guidelines[15,19]. The 'how' of change is captured in the detailed presentation of clear communication strategies that should enable clinicians to assess patients' unvoiced agendas and identify and respond to information needs[20].

### The STAR trial

The primary objective of the trial is to determine whether clinicians' exposure to the STAR Educational Program results in fewer antibiotics being dispensed to the practice's patients during the year following completion of the intervention. This is to be assessed using routinely gathered data such as Prescribed Audit Reports and Prescribing Catalogues (PARC).

Process evaluation of clinical trials serves i) to investigate intervention delivery fidelity, ii) to evaluate the feasibility and efficacy of the program in daily practice, and iii) to identify areas for intervention refinement[21]. The overall STAR trial process evaluation is multi-faceted and includes the views of participants from the experimental group, seminar facilitators' views, practice background information, mapping of participants' use of the online learning program (e.g., log-in times and duration, or whether access takes place during working or leisure hours), STAR web forum contributions commenting on process and program content, and detailed economic evaluation. Here we focus mainly on participants' views regarding their engagement with the STAR Program but, where relevant, we also refer to other components of the process evaluation data.

The perspectives of participants are essential to understanding if and how the STAR Program has contributed to behaviour change. A focus on how particular issues are voiced, in combination with the mapping of certain themes or patterns of thought, will provide a representative overview of perceived advantages and disadvantages of the program, as well as identify potential areas for improvement. Moreover, clinicians' perceptions of patient behaviour and attitudes in relation to changes in their own behaviour will give an indication of the efficacy of the program in practice.

### The STAR Educational Program

**Figure 1:**
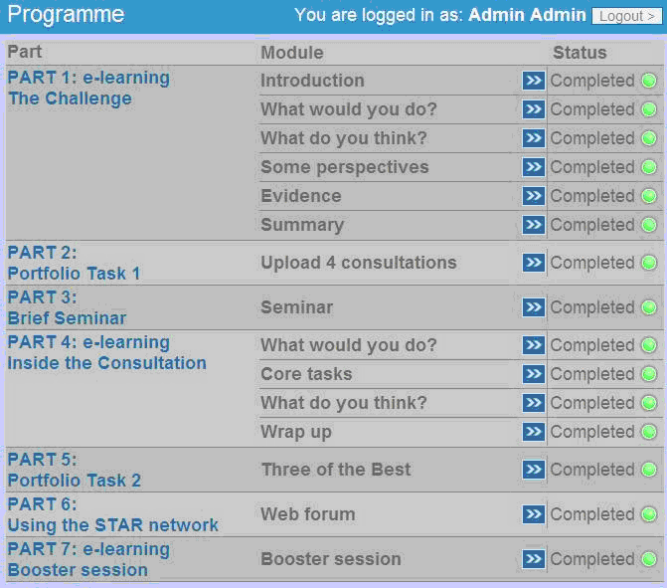
**Overview of the STAR Educational Program**.

The STAR Program consists of five core parts, supplemented with an ongoing web forum (part 6), and a booster session (part 7) provided approximately six months after completion of the core program (see Figure 1). Parts 1 and 2 involve an online introduction to the topic of antibiotic resistance and prescribing, and probe participants' own views on this issue while also providing case scenarios for reflection and examples of the latest evidence in the form of reference charts and summarised readings. The aim here is to heighten awareness and encourage clinicians to consider how they manage common infections in practice. Part 3 of the program is an on-site, face-to-face seminar in which practice prescribers meet with a STAR study trainer who facilitates discussion about practice-unique prescribing data, and resistance data measured from samples submitted by the practice over a five to ten year period prior to the study. Part 4 uses video scenarios to demonstrate key consultation strategies, illustrating in detail how 'core tasks' such as 'lifting the lid', 'information exchange', and 'wrap up' can be used to gain a better understanding of patients' concerns, expectations, and attitudes. In part 5 this learning experience is consolidated by asking clinicians to describe and reflect on three examples from their own clinical practice. Although at the time of the process evaluation interviews the web forum was accessible to all intervention group participants, the booster session had not yet been provided.

## Methods

Sixty-eight practices agreed to participate in the trial. As two practices withdrew from the study after randomisation, the intervention group consisted of 33 practices. Thirty GPs and one Nurse Practitioner from this group participated in semi-structured interviews, with one practice being represented by both a Nurse Practitioner and a GP. Participants were purposively sampled for gender, experience (0-10 years (L), 10-20 years (M), >20 years since date of qualification (H)), level of antibiotic prescribing in the practice as evident from pre-trial data (ranked high or low according to whether or not practices were in the top 50 percent of prescribing rates), and training completion (within 0~8 weeks from seminar; 9~18 weeks from seminar or not yet completed at time of sampling).

Participants were initially approached by letter, they were made aware that the interviews were part of the process evaluation of the trial and that they would be conducted by a member of the STAR team. Not all STAR participants initially approached were able to take part in an interview and so alternatives were approached according to the purposive sampling frame (see Table 1).

**Table 1:** Overview interviewee sampling.

	All participants (127)	Originally sampled (34)	Actually interviewed (31)
**Gender**			

Female	38.40%	39.39%	35.48%

Male	61.60%	60.61%	64.52%

**Total**	100%	100%	100%

			

**Experience**			

0-10 yrs	35.20%	36.36%	32.26%

11-20 yrs	35.20%	33.33%	35.48%

> 20 yrs	26.40%	27.27%	29.03%

Nurse Practitioner	1.60%	3.03%	3.23%

Not known	1.60%	0.00%	0.00%

**Total**	100%	100%	100%

			

**Prescribing**			

HIGH	32.80%	45.45%	48.39%

LOW	67.20%	54.55%	51.61%

**Total**	100%	100%	100%

			

**Training completion**			

Early	28.80%	36.36%	25.81%

Late	71.20%	63.64%	74.19%

**Total**	100%	100%	100%

Interview questions covered three main areas: 1) general information about practice location, time worked in the current practice and level of importance given to the issue of antibiotic resistance before taking part in STAR, 2) effects of the program as perceived by participants after completion of parts one to six, and 3) detailed evaluation of presentation, content, and structure of the actual learning program (see Appendix A). Interviews were conducted by telephone and digitally recorded. They ranged from ten to thirty-six minutes, with an average duration of twenty-five minutes. Data was transcribed verbatim and transcripts coded using NVivo qualitative analysis software. The main coding categories reflected the questions asked during the interview, with deeper level coding focusing on classification of interviewee contributions, as well as positive or negative stance towards topics raised.

To ensure validity of categorisation, thirty percent of transcribed interviews were independently examined by two qualitative researchers on the STAR team, disagreements were discussed and consensus regarding content and labelling of coding categories was reached. The data was analysed using thematic content analysis in which outcomes reflect emerging trends in the data as evident from the prevalence of particular categories and the reiteration of particular points of view.

The STAR study was approved by the Multi-centre Research Ethics Committee (MREC06/MRE09/31) and all Local Health Boards in Wales.

### Transcription conventions

In the selected data extracts, for ease of understanding, verbatim speech has been minimally adjusted and grammatical conventions were adhered to. The following transcription conventions were applied:

[word]      text added for clarity

(word)      transcriber's guess at unclear speech

((word))    transcriber's description of non-speech sounds

...             text not directly relevant to topic omitted from original transcript

word-       truncated speech; rapid switch to new formulation

*word         *spoken with added emphasis

## Results

### Perceptions of main changes to clinical practice

#### Awareness and behaviour

Participation in the STAR Educational Program was presented as reinforcing already existing knowledge:

It has reinforced [my views on prescribing antibiotics] more than anything and perhaps has improved my use of a wait and see policy rather than actively prescribing. I think as a practice we were relatively low prescribers anyway but it was nice to reinforce that and to make sure what we're doing is correct really. (GP142)

In particular, locally relevant data was seen as reinforcing the salience of the issue of antibiotic resistance:

I think it is easy to get blasé about antibiotic resistance and I think having the study has focused my mind and having local knowledge and the summary of the sort of changes that might come about if you concentrate on it [is] helpful; I think it raises the profile of the whole subject a long way. (GP185)

Interviewees also reported greater self-confidence in implementing their prescribing decisions:

It has given me a lot more confidence in refusing antibiotics and in fact I have been surprised at how little resistance I've had when I say I won't give something and they say that's good. (GP271)

Inevitably, participants varied in the manner in which they expressed this change in awareness and self-confidence, the positive accounts above contrasting with the two more dismissive statements below.

In the following excerpt, the interviewer (I) probed the GP about whether participating in STAR influenced his antibiotic prescribing, and the GP seemed quick to point to minimal effect:

I: so would you say that [STAR] has changed your actual prescribing behaviour?

GP: u::hm just reminded me let's put it that way

I: mhm mhm

GP: it has just reminded me of the vigilance and how important it is you know not to prescribe when it is not necessary and things

(GP 199)

Similarly, in the following example the GP put forward that STAR was only one of the components that characterised already ongoing behaviour modifications:

I think ... STAR has come along in a general progressive movement in my prescribing which is going from prescribing obviously more to prescribing gradually less and a gradual education of my patients towards that and I see it more that- it's actually been another impetus along the direction that [I] was already travelling and so ... it has encouraged me in giving the sort of confidence and the backing to continue to move in that sort of direction; it's something we were already doing very much. (GP 229)

Whereas the interviewees in the earlier three examples ascribed a clear agency to the STAR Program: 'It has reinforced'; 'it raises the profile'; 'It has given me more confidence' and the last two excerpts relegate STAR to a more secondary position: 'it just reminded me'; 'STAR has come along in (an already) progressive movement'; 'it's been *another *impetus', all five data excerpts acknowledge the positive effect of heightened awareness. That there should be individual differences was only to be expected as, after all, the degree to which antibiotic prescribing behaviour needed to be modified varied as well.

#### Enhanced communication versus consultation length

Generally, participants felt that applying the communication skills presented in STAR provided greater insight into patients' wishes and demands, which was then seen to impact directly on future consultation rates and, ultimately, on patients self-managing future episodes:

I think [my patients] probably get a better deal out of me actually ... because they get more time which is most of all what they're after and actually I mean I never used to leap on the idea of antibiotics as a way of terminating the consultation quickly before anyway but I think I'm just a little bit more prepared to listen to the patient's experience ... more so than I was ever used to doing. (GP 150)

Most clinicians perceived consultations to be lengthened by an average of two to three minutes as a result of implementing the new communication skills. However, again this was generally seen as a positive trade-off to reduce or prevent future consultations:

I think it probably does make the consultations a bit longer but not drastically so you know and I would think I feel reasonably confident that we will you know recoup that extra time in the future by people hopefully not re-presenting for antibiotics quite as often. (GP 216)

I suppose in the long term they won't come back will they so in a way it's saved you time ... next time perhaps they'll not come in because you get people coming in saying 'I've had a cough for two days' and you think 'well, so what, bugger off', whereas if you'd spend more time on the first consultation explaining to them why you think they don't need them they may not come back, but it's certainly in the short term that it takes more time. (GP 256)

#### Impact on (the) practice

Although the type of antibiotics prescribed was not an explicit interview question (see Appendix A), the topic did arise tangentially, as in the following two data extracts:

... my perception is that I am using [antibiotics] less often, and I've changed the range of antibiotics that I commonly use. (GP 275)

... occasionally I have had a patient who has had severe reactions, for example, when a patient had really bad jaundice after some amoxicillin so it is just a perception from my viewpoint but I seem to see the incidence as far as side effects or bad reactions is less than it was and whether that is related to prescribing more appropriately or less, I don't know. (GP 207)

However, as GPs reported increased self-confidence regarding prescribing decisions, to a large extent based on the research evidence provided in the online learning program, the general references to 'more appropriate' prescribing behaviour in the data may well indicate a shift towards narrow spectrum antibiotics prescription in addition to prescribing fewer antibiotics overall.

There was little perceived change in the frequency with which specimens were sent to the lab, nor was there a noticeable increase in re-consultation rates, although in this respect, the need to present a united front across the practice was repeatedly mentioned:

... colleagues have a few patients who according to criteria were not prescribed at the time but they re-present a week later and their condition is worse therefore I have prescribed. But if they come and there is no change I don't prescribe. I mean you have to reinforce what they were told last time. I think we tried to do that more since the program, not just give in to have a quiet life. (GP207)

... we have one mum who brought a child for 3 days on the trot; really struggled with the concept of having no antibiotics and we all, the thing was we could see her coming in the end, ... and we were communicating amongst ourselves to make sure we didn't actually break or crack under the pressure. (GP 161)

Finally, two GPs stated that neither their thinking nor their prescribing behaviour was in any way influenced by STAR, although both of them indicated this was because they were already implementing the skills promoted in the STAR Program. As one of them summarised:

... it was a revision exercise, I think, ... if you do a study and you say 'oh, I'm already doing that', at least you know that what you are doing is right ... the reason that [participating in STAR] did not change my practice is not because I ignore what was being said, it was because I had already agreed with what was being said. (GP 171)

### Evaluating the STAR Educational Program

#### Communication skills examples

In evaluating the contents of the program, rather than its reported effects, views were sometimes polarised. The presentation of key communication skills was described either in terms of new, useful and exciting, or as old and familiar, though perhaps in need of 'brushing up'. In either instance, however, implementing these skills was acknowledged as leading to better patient care and, ultimately, greater personal satisfaction:

I think the communication skills aspect was good, being able to ask patients what they feel about antibiotics and to have a more adult conversation about it ... it sort of encourages you not just to be defensive and [say] 'we don't want to prescribe' but be proactive ... asking the patients what they felt was the benefit of taking antibiotics and what did they think they were going to get out of it .... and sort of telling people it is a self-limiting illness, some of those skills I thought was very good and make it much easier to prescribe the way I'd like to. (GP 229)

#### The seminar

Respondents viewed the seminar as providing a much-needed 'human touch', although a small number of interviewees, especially those working in single-handed or very small practices, considered it a waste of time and money. Participation in the program seminar was also presented as a unique chance to focus on a particular issue and to increase communication within the practice team:

... the trouble is in general practice you don't have time to sit and talk and [it's] usually sort of a business practice meeting we don't often have clinical sort of where we actually discuss and necessarily change or discuss the pros and cons of various things on a regular basis. I'm not saying we don't ever talk about things at all, we communicate quite well, but it's finding the time to do it. (GP 161)

Seminar trainer feedback indicated that it proved difficult at times to gather all the trial participants from a particular practice at a particular time, with the absence of practice nurses, who are often in charge of minor illness (telephone) triage, especially commented upon. Overall, however, trainers described the seminar discussions as lively, with participants most eagerly engaged in discussing local resistance rates as correlated with own practice data.

#### The online training

The online aspect of the training was generally evaluated positively, with a particular emphasis on its promotion of independent learning and flexibility in accessing the program. However, six out of thirty-three practices experienced (initial) technical difficulties and especially in practices with older computer systems, or for clinicians less comfortable with IT, delays in video streaming and inability to access the program depending on certain computer settings could lead to frustration:

... there was a kind of pointlessness about the use of the technology, having video streaming that just made it irritatingly slow to download and it didn't contribute anything, and you'd actually watch a videotape of somebody *talking*, I would just as soon have *read *the text to be honest. (GP 171)

Finally, some participants found the video material lacking in authenticity:

... there was some amusement during the video consultation with the various patients and doctor scenarios because it all seemed to go so beautifully according to plan and the patients never argued and there was lots of time and I thought - we all discussed that and we thought it was rather amusing, we didn't think it was totally realistic. (GP 207)

#### Research evidence and guidelines

The presentation of up-to-date evidence was generally seen as one of the most useful aspects of the STAR Program. Participants described how they discussed the modified Centor clinical scoring tool for managing sore throat, as well as the prescribing guidelines and evidence summaries with patients during consultations:

... you gave us guidelines on- primary care guidelines that have been very useful actually. Again, we've given our nurses copies of those to have a look at when they are seeing patients with minor illnesses. You know, I think no one has given them training in good antibiotic prescribing so I do think they over-prescribe, even though they're very good. I think those guidelines have been quite helpful, in fact we keep them pinned up by the uh, when they're doing nurse triage we keep them pinned up by the phone, so they can refer to those. (GP 248)

... the Centor guidelines, the other guidelines, can't remember what they were called now, the ones for the sinusitis and things you know, those I actually have them on my desktop. So what I do is I just put them on if I get someone stroppy ... just put them on and turn the screen and say 'read that, that's the guidelines we've got', because if you've given them an examination and you know they haven't got a temperature and they haven't many chest signs ... On the whole they tend to sort of 'oh okay' then, it's on the screen so it must be true and they see that's it's, you know, it's an official document. (GP 256)

As evident from seminar trainer feedback as well as interview data, and in line with the 'computer-says-no' scenario presented in the above data excerpt, STAR participants repeatedly requested antibiotic resistance information leaflets or posters that can be displayed in surgery waiting rooms. Interviewees noted that presentation of the research evidence and guidelines in this more generally accessible format could have provided them with an added tool, and they expected it to be part of the overall program.

#### Case studies and self-reflection

About a fifth of interviewees reported that they did not see the merits of the reflective exercises or recording their own consultations online:

I: ... and what did you think was the least useful

GP: I think finding my own cases to put in. I don't know there's plenty of cases you could have found. It was hard to find an interesting one. But in terms of looking at that it didn't really affect what I was doing in any way, it was just a bit time consuming. That was a bit of a chore. (GP152)

However, one of these participants, unprompted, addressed his own reservations on this issue, thereby aligning himself with the majority standpoint:

... the tasks of recording some of one's own consultations ... I don't know whether recording them had any benefit over simply thinking about them. Obviously recording them takes up a bit more time, but having said that I don't know if I didn't have to record them whether I'd really spend time ((laughs)) thinking about (those cases). It felt frustrating at some level but I'm well aware that that sort of thing does actually improve one's processing of it. (GP216)

#### Overall evaluation of key STAR components

The core aspects of the STAR Program considered 'most useful' and reported by these sampled participants as responsible for influencing their prescribing most were the research evidence and guidelines provided in the program, and the online communication skills examples, both of which were explicitly mentioned by 12 of the 31 interviewees. Ten interviewees reported that their prescribing behaviour had changed because of the increased overall awareness of the antibiotic resistance issue that results from working through the program as a whole, with four of those singling out the impact of discussing local resistance rates during the STAR seminar.

In contrast, there were also respondents who considered the research evidence not directly relevant to their own clinical practice, or found it too difficult to process online. Moreover, the web forum, originally envisaged to become an ongoing learning resource, was dismissed by many as irrelevant, a format participants could not or would not engage with, even if their busy working lives would allow them time to do so.

However, it was clear that all interviewed study participants subscribed to the view summarised by GP 207 as follows:

... overall I think it's just the being better educated and having therefore more clinical expertise and [the] communication tools to prescribe appropriately, treat appropriately, and therefore give better patient care, which is the bottom line. (GP207)

## Discussion

### Summary of main findings

Most of the participants put forward that the STAR Educational Program increased their awareness of the problems of antibiotic resistance as well as how their management of common infections might be improved. GPs recognised the value of enhanced communication skills, both as a means to provide better patient care and as a route to greater professional satisfaction. Using these skills in combination with the up-to-date evidence to exchange information with their patients about the use and the effects of antibiotics was recognised as having both long-term and widespread impact and there was an expressed willingness to invest time and energy in achieving this goal. GPs felt that the STAR Program provided them with the tools to negotiate with patients about antibiotics in the best possible interests not only of individual patients but also, in the long run, the population at large. Besides addressing the issue of antibiotic resistance, applying the communication skills was presented as enhancing doctor-patient rapport. In the practice as a whole, taking part in the STAR Educational Program could lead to a joint focus on straightforward and achievable goals, drawing together practice partners and their associates, including doctors in training. The GPs and Nurse Practitioners felt empowered by their increased insight, even if in some cases this simply meant the confirmation of views already held.

Not all aspects of the STAR Program were consistently judged positively. Technical difficulties clearly influenced the appreciation of the online video presentations and contents and presentation of the generally valued research evidence was also criticised. Trawling through patient notes for representative case studies to enter online as part of the STAR core tasks was seen as a time-consuming activity with little practical benefit, while similar views were expressed about participating in the web forum discussions. However, although individual participants may have criticised individual components of the program, overall it was evaluated positively.

### Study strengths

One of the strengths of this interview study is that it forms only part of the overall STAR trial process evaluation and yet it touches upon aspects of all the other process evaluation components, unifying these potentially disparate elements in the obvious relevance they all bear to how clinicians manage to put the STAR Educational Program into practice. Interviewees made reference to the cost of the seminars or the study as a whole, they commented on seminar content and delivery in a way that overlapped with seminar trainer feedback, they volunteered information about the location and detailed make-up of their practice and how this might influence antibiotic prescribing, and gave insight into when, where and how they accessed the online training. By making all these issues explicit, interviewees illustrated how the combined STAR trial process evaluation components can contribute to providing insights into the efficacy of the learning program and the fidelity of its delivery. In this way the interview study in itself becomes an indicator of the feasibility of a fundamental trial protocol component.

GPs' generally heavy workload makes it difficult to reserve time for what are essentially non-core activities such as taking part in research related interviews. To ensure that the widest possible range of opinions could be obtained, the STAR team selected clinicians from each of the thirty-three intervention group practices across the four key sampling domains (gender, experience, prescribing, program completion). However, initial invitations to be interviewed were sometimes declined which meant that alternatives had to be sought. It is another strength of this study that in spite of these practical difficulties, the sampling criteria were just as rigorously applied for second or third interviewee choices. Only three clinicians who originally consented to be interviewed declined to take part after all.

A final strength of allowing this group of participants to elaborate on their experiences with STAR is that it not only provided insight into what they considered the most effective aspects of the learning program, but also where they saw areas for improvement. The interview schedule included specific questions inviting talk about positive as well as negative experiences and asked interviewees to elaborate on any additions or changes they might want to make.

### Study limitations

When using a semi-structured interview format, to a great extent the questions answered are the questions asked. In general, this semi-rigid structuring prevents frequent asides and more individual comments and may prevent topics from arising that could have contributed to greater insight into how effective the learning program may be. This is certainly a limitation, but perhaps a necessary one in an interview in which so many different aspects of a complex learning method need to be addressed.

It is clear that in many cases the interviews took place too long after the STAR Program had been finished as some interviewees simply could not remember specific details of program contents or how it was experienced at the time. However, lack of recall only appeared to influence views in one or two instances and did not prevent interviewees from expressing views on their experience as a whole.

Another limitation is that not all participants from the intervention group could be interviewed, although the sampling procedure was designed to select interviewees who would best represent the main sampling criteria. However, we are aware that, potentially, this selection process could have introduced bias as there is a possibility that invited participants declined to be interviewed because they felt uncomfortable about expressing any negative views to a member of the STAR team. Moreover, due to the timing of the intervention, it was impossible to obtain the views from clinicians in the control arm of the study who were offered the STAR Educational Program at the end of the study.

### Comparison with existing literature

Participants in the STAR Program reported that the intervention increased their awareness and that they found the up-to-date evidence useful in making decisions about the best treatment for patients, as well as increasing their confidence. Previous studies have used educational approaches with some success to try and reduce inappropriate antibiotic prescribing[22], although when these approaches were implemented on a larger scale, the authors did not find a reduction in prescribing rates[23]. However, clinicians in that study specifically highlighted the importance of appropriate communication skills during consultations for common infections; they felt it gave them greater insight into patients' needs and wishes. A more recent study, which focused on improving communication in particular, achieved an impressive 40 percent relative reduction in antibiotic prescribing at 12 months[24]. In evaluating the STAR Program, interviewees also emphasised the importance of communication skills as they felt it is easy to misjudge patients' expectations by assuming patients want antibiotics when in reality they want to be listened to, examined properly and appropriately reassured[8,25,26].

### Implications for future research and clinical practice

These interviews, with their predominantly positive response to the program, illustrate that clinicians find this particular method of Continuing Medical Education (CME) useful, flexible and often enjoyable. The positive feedback regarding the face-to-face element (the seminar) suggests that this was an important part of the learning program. This type of 'blended' approach (e-learning plus face-to-face) may provide a cost-effective way to deliver CME to clinicians. In 1999-2000 in the UK about £1bn was spend on CME[27]. The most common barriers to completing CME are cost of the education, loss of income, family commitments and time[28,29]. The effectiveness of traditional CME delivery formats, for example conferences, has been questioned[30]. Online learning may be a better alternative, since it is more flexible and cost-effective to deliver. The evidence base for the effectiveness of e-learning or blended learning approaches is limited by the methodological quality of studies. However, there is some evidence that e-learning or blended learning approaches improve knowledge and are at least as good as traditional courses [31-35].

## Conclusions

As is evident from the interview data, working through the STAR Educational Program can lead to a greater awareness of the problem of antibiotic resistance, increased self-confidence, and a change in prescribing behaviour for some. Participants put forward that being better informed and having learned or refreshed specific communication skills contributed to a perceived reduction in antibiotic prescribing and a better understanding of patients' expectations.

Apart from some initial technical problems, interviewees did not report any major difficulties in accessing the program or in implementing any of the changes it promotes and, in fact, recommended making STAR available for teaching medical students, for clinicians working in Out of Hours services and for Nurse Practitioners who are often the first point of contact in minor illness cases.

Suggestions for improvement include re-thinking the scenarios for the communication skills videos to better reflect real-life consultations, providing patient information leaflets or posters, and more clearly explaining the purpose of the reflective exercises and the case studies that participants are asked to add online. In addition, where possible, the technical platform may be in need of some adjustment to allow smoother transition through the program in cases where people do not have access to high-speed broadband or fast computers.

The participants interviewed for this study judged the content of the STAR Program, as well as the delivery methods as timely, necessary and feasible. Based on their experiences in clinical practice, some of the interviewees recommend roll-out of the program not only across a wider geographical area but also across a wider range of medical disciplines and care delivery contexts.

## Competing interests

The authors declare that they have no competing interests.

## Authors' contributions

MJB led the writing of the manuscript, conducted the interviews and was responsible for transcribing and analysing the data. SS served as second data coder and contributed substantially to the discussion section of the paper. CB is the principal investigator on the STAR trial, he commented extensively on earlier versions of the paper. KH, FD, MH and JE also made valuable suggestions for improvement. All authors contributed to, read and approved the final version of the manuscript.

## Appendix A: Full interview schedule

### STAR Process Evaluation: Interview Guide

#### 1. General

▪ confirm name

▪ length of time in the practice

▪ rural, urban, valleys practice?

▪ what level of importance given to *antibiotics resistance issue *before training: low, medium, high?

#### 2. STAR participation

▪ what were your expectations of time commitment before you started? how did this work out in practice? (can you give an estimate of the total time it took to complete the learning program?)

▪ how did you experience the cooperation with the STAR team? did you communicate directly or via the practice manager?

#### 3. Main questions

(i) How could STAR *content *be improved?

(ii) How could STAR *training *be improved?

#### **Prompts:**

##### **a. impact of STAR Educational Program**

▪ has taking part in the program changed *your views *on prescribing antibiotics? has it changed your actual *prescribing behaviour *in any way?

▪ do you think patients have noticed a difference pre-STAR and post-STAR?

▪ do you feel that using the skills promoted in the STAR Program affects the length and the nature of the consultation? how?

▪ is it possible to estimate (in minutes) the extent of a change in length?

▪ what is the perceived impact of taking part in STAR on the practice as a whole? (e.g., did STAR participation lead to formal/informal discussions about antibiotics prescribing in the practice)

▪ do you think there are any barriers to implementing the communication skills promoted in STAR?

▪ perception of whether patients will use other means to get antibiotics? (OOH, see a different GP in the practice, etc)

▪ what is your *perception *of whether patients will be re-consulting more often

▪ do you use delayed prescribing as a technique for reducing prescribing?

▪ if you use delayed prescribing, what is the procedure for delayed prescribing in your practice? (e.g., during the consultation or do patients collect prescription from reception a few days later?)

▪ has taking part in the STAR Program had any influence in how often you send a specimen to the lab?

##### **b. STAR seminar**

▪ general impression

▪ is the seminar necessary?

▪ length of seminar

▪ suitability of venue and trainers

▪ seminar contents

▪ impact of seminar

▪ suggestions for improvement

##### **c. STAR software**

▪ general impression

▪ using the software (flow, navigation of learning plan)

▪ how useful was the information, both the actual program and the resources provided

▪ ratio video - text - reflective exercises?

▪ any problems with software?

▪ suggestions for improvement?

##### **d. Completion of training**

▪ where there any particular 'stumbling blocks'?

▪ have you gone back to software since completing the program?

▪ was the training provided in the program enough?

##### **e. STAR web forum**

▪ have you already used the web forum?

▪ expectations of web forum?

▪ any comments/suggestions for use web forum?

▪ are you likely to keep using the web forum in the future?

▪ are you aware that literature updates will be available?

▪ are you aware that STAR team will keep forum updated with questions arising from latest research?

#### 4. Summary questions

▪ **What aspects of the STAR Program were most useful for you?**

▪ **What aspects of the STAR Program were least useful for you?**

▪ **Which aspects of the STAR Program have influenced your prescribing most?**

#### 5. Finally

▪ **What made you decide to participate in the STAR study?**

## Pre-publication history

The pre-publication history for this paper can be accessed here:

http://www.biomedcentral.com/1471-2296/11/34/prepub
